# Microbiota dysbiosis in odontogenic rhinosinusitis and its association with anaerobic bacteria

**DOI:** 10.1038/s41598-022-24921-z

**Published:** 2022-12-05

**Authors:** Yen-Ting Lu, Shao-Hung Wang, Ming-Li Liou, Cheng-Yang Lee, Yu-Xuan Li, Ying-Chou Lu, Chung-Han Hsin, Shun-Fa Yang, Yih-Yuan Chen, Tzu-Hao Chang

**Affiliations:** 1 Institute of Medicine, Chung Shan Medical University, Taichung, Taiwan; 2 Department of Otolaryngology, St. Martin De Porres Hospital, Chiayi, Taiwan; 3 Department of Otolaryngology, Chung Shan Medical University Hospital, Taichung, Taiwan; 4 School of Medicine, Chung Shan Medical University, Taichung, Taiwan; 5 Department of Microbiology, Immunology and Biopharmaceuticals, National Chiayi University, Chiayi, Taiwan; 6 Department of Medical Laboratory Science and Biotechnology, Yuanpei University, Hsinchu City, Taiwan; 7 Office of Information Technology, Taipei Medical University, Taipei City, Taiwan; 8 Department of Medical Research, Chung Shan Medical University Hospital, Taichung, Taiwan; 9 Department of Biochemical Science and Technology, National Chiayi University, Chiayi, Taiwan; 10 Clinical Big Data Research Center, Taipei Medical University Hospital, Wu-Hsing Street, Taipei City, 110 Taiwan; 11 Graduate Institute of Biomedical Informatics, College of Medical Science and Technology, Taipei Medical University, Taipei, Taiwan

**Keywords:** Microbiology, Diseases, Health care, Medical research, Risk factors, Signs and symptoms

## Abstract

Odontogenic rhinosinusitis is a subtype of rhinosinusitis associated with dental infection or dental procedures and has special bacteriologic features. Previous research on the bacteriologic features of odontogenic rhinosinusitis has mainly used culture-dependent methods. The variation of microbiota between odontogenic and nonodontogenic rhinosinusitis as well as the interplay between the involved bacteria have not been explored. Therefore, we enrolled eight odontogenic rhinosinusitis cases and twenty nonodontogenic rhinosinusitis cases to analyze bacterial microbiota through 16S rRNA sequencing. Significant differences were revealed by the Shannon diversity index (Wilcoxon test *p* = 0.0003) and PERMANOVA test based on weighted UniFrac distance (Wilcoxon test *p* = 0.001) between odontogenic and nonodontogenic samples. Anaerobic bacteria such as *Porphyromonas, Fusobacterium,* and *Prevotella* were significantly dominant in the odontogenic rhinosinusitis group. Remarkably, a correlation between different bacteria was also revealed by Pearson’s correlation. *Staphylococcus* was highly positively associated with *Corynebacterium*, whereas *Fusobacterium* was highly negatively correlated with *Prophyromonas*. According to our results, the microbiota in odontogenic rhinosinusitis, predominantly anaerobic bacteria, was significantly different from that in nonodontogenic rhinosinusitis, and the interplay between specific bacteria may a major cause of this subtype of rhinosinusitis.

## Introduction

Odontogenic rhinosinusitis is an important but underrecognized subtype of rhinosinusitis; up to 10–30% of maxillary sinusitis cases have odontogenic causes^1–3^. This subtype of rhinosinusitis also has unique radiographic, microbiologic, and clinical features, indicating its dental origins^4^. Unlike most rhinosinusitis cases, which are bilateral and diffuse, odontogenic rhinosinusitis is mainly unilateral and localized. For example, in a previous study, 75% of odontogenic rhinosinusitis cases were unilateral and limited to the maxillary sinus^2,5^. However, the exact cause of this localization remains unknown. Previous studies have implicated microbes as being responsible for inducing local inflammation, leading to increased mucosal permeability^6^. Microbes could play a role in the formation of odontogenic rhinosinusitis. Therefore, further research on localized microbiota is critical to understanding odontogenic rhinosinusitis.

The bacteriology of odontogenic rhinosinusitis is distinct from that of nonodontogenic rhinosinusitis^4^. Previous studies using bacterial culture methods have reported a polymicrobial etiology in odontogenic rhinosinusitis, with a predominance of anaerobic organisms^7,8^, particularly *Peptostreptococcus*, *Prevotella,* and *Fusobacterium*^7–10^. Mixed aerobic and anaerobic infections have also been well documented^4^. For example, Zirk et al. observed 70% and 30% incidence rates of anaerobes and aerobes, respectively, in odontogenic rhinosinusitis^11^. Although the critical role of anaerobes and the polymicrobial bacteriology of odontogenic rhinosinusitis are well established, the microbiota difference between odontogenic and nonodontogenic rhinosinusitis and the association and correlation between specific bacteria are difficult to determine using culture-dependent methods. Saibene et al. reported that 60% of control samples failed to yield any bacterial growth on culture^1^. Therefore, in this study, a culture-independent approach involving 16S rRNA gene amplicon sequencing was used to compare the microbiota composition between odontogenic and nonodontogenic rhinosinusitis cases and to determine the relationship between the involved bacteria.

## Methods

### Study design and population

We conducted a prospective cohort study of patients with chronic rhinosinusitis (CRS), including those with odontogenic or nonodontogenic rhinosinusitis. The patients had all undergone functional endoscopic sinus surgery (FESS). All patients were older than 20 years and had been diagnosed with CRS according to the American Rhinologic Society criteria^12^. Sinus inflammation was revealed by nasal endoscopy, and sinus opacification was confirmed by computed tomography (CT)^13^. Patients who had sinonasal malignancy or cystic fibrosis, were immunocompromised, had received systemic or topical antibiotics/antifungal medication within 1 month before surgery, or reported pregnancy were excluded^14,15^. The remaining patients were then grouped according to CT findings into odontogenic and nonodontogenic rhinosinusitis groups^4,13^. Patients with CT features indicating periapical radiolucency and opacification over the ipsilateral sinus at least including the maxillary sinus were defined as having odontogenic rhinosinusitis, which was confirmed by a radiologist, otolaryngologist, and dentist^4,13^. Demographic data, as well as endoscopic and radiologic features, were recorded and analyzed.

### Ethics statement

This study was approved by the Human Ethics Committee of St. Martin De Porres Hospital, Taiwan (Approval No. 20B-003) and the informed consent was confirmed by every involving patient. All methods were performed in accordance with the relevant guidelines and regulations.

### Sample collection

Swabs were collected from the middle meatus and rotated at least 5 times under endoscopic guidance during FESS. All procedures (including sample collection and FESS) were performed by the same surgeon (Y.-T.L.). After swabs were collected, they were placed in a sterile container on ice for immediate transport to the laboratory and were subsequently stored at − 80 °C until DNA extraction^15^.

### DNA extraction

The extraction method followed that of our previous studies^15,16^. A Qiagen Blood Mini Kit was used according to the manufacturer’s instructions. Purified DNA was eluted, quantified, and stored at − 20 °C.

### 16S metagenomics sequencing

The V3–V4 region of 16S rRNA gene was selected and amplified according to the method in our previous studies using V3 5′-CCTACGGGGNGGCWGCAG-3′ and V4 5′-GACTACHVGGGTATCTAATCC-3′ primers^15,16^. Raw, paired-end reads were trimmed of poor-quality reads with the criteria of Phred quality score Q < 20 and length less than 200 bp before further analysis.

### Microbial community analysis and statistical analysis

QIIME2 was used to analyze amplicon sequences as well as for sample demultiplexing, reads denoising, sample and sequence filtering through a feature table, alpha and beta diversity estimation, and taxonomy analysis. Reads denoising was conducted using the QIIME2 dada2 plug-in module, which infers true sequencing reads by correcting amplicon sequencing variants, truncates the length of the 3′ and 5′ ends of the reads, assesses read quality, and removes chimeric reads. After denoising, the feature table was exported, and the samples, sequences, and features were filtered using the feature table according to the following criteria: (1) for each sample, the total number of reads was > 10,000, and (2) for each feature, the total number of reads was > 50. Alpha diversity was calculated using the Shannon diversity index, and the Wilcoxon test was used to compare alpha diversity between the groups. Beta diversity was calculated using the weighted UniFrac distance, and microbiome communities from different samples were visualized using principal coordinate analysis (PCoA). For taxonomy assignment, SILVA v138 genomic databases were used as the reference for bacteria. We used the R package DESeq2 to calculate differentially abundant microbiomes (DAMs) between odontogenic and nonodontogenic samples by using feature tables with different taxonomy levels. PICRUSt2^17^ was applied to infer the functional content of the microbiome based on OTU, and STAMP^18^ was used to calculate and plot the function pathways and statistical results.

## Results

### Clinical characteristics of the patients

This study included 8 and 20 patients with odontogenic and nonodontogenic CRS, respectively, and Table 1 presents their demographic data. Eight patients were assigned to the odontogenic rhinosinusitis group, because they presented with periapical radiolucency lesions, as confirmed by CT. No significant differences with regard to asthma, diabetes mellitus (DM) or smoking history were found between these two groups. Clinically, the extent of odontogenic rhinosinusitis was limited compared with that of nonodontogenic rhinosinusitis. In all eight patients, odontogenic rhinosinusitis was unilateral and mostly limited to the maxillary sinus, but in eleven of twenty patients, nonodontogenic rhinosinusitis presented as bilateral lesions with multi-sinus inflammation.

**Table 1:** Demographics of patients.

	Odontogenic (n = 8)	Non-odontogenic (n = 20)	*p* value
Gender (M : F)	6:2	13:7	0.609
Asthma	0	0	NA
DM	1	1	0.486
Smoking	3	2	0.533
**Endoscope features**			
Purulence	8	7	0.302
Polyp	2	6	0.132
Unilateral/bilateral	8/0	9/11	0.002*
**CT features**			
Periapical radiolucency	8	0	0.000*

*DM* diabetes mellitus, *CT* computed tomography.

*p < 0.05.

### Microbial features and microbial diversity between odontogenic and non-odontogenic rhinosinusitis

A total of 28 nasal samples were subjected to demultiplexing and denoising, following which the corresponding 16S dataset was obtained, with a mean of 37,791 reads per sample and 1343 features. Subsequently, 320 bacterial taxa at the genus level were annotated according to the SILVA database.

**Figure 1:**
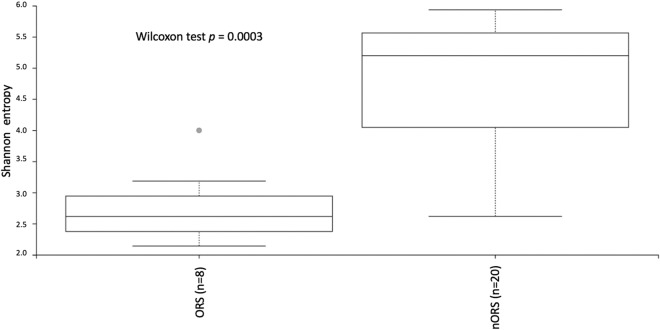
Shannon diversity (alpha diversity) between microbiomes collected from lesion sites in odontogenic and nonodontogenic rhinosinusitis samples, as revealed by 16SrRNA sequencing (Wilcoxon test *p* = 0.0003). ORS: odontogenic rhinosinusitis; nORS: nonodontogenic rhinosinusitis.

There was no statistically significant difference with regard to the total number of reads of 16S rRNA gene between odontogenic and nonodontogenic groups, indicating that these two groups of patients had similar total bacterial load. However, the sinonasal microbiota of showed significantly difference and revealed decreased bacterial diversity in odontogenic groups when compared with the nonodontogenic group. The Shannon diversity index in the odontogenic rhinosinusitis samples was significantly lower than that in the nonodontogenic rhinosinusitis samples (Wilcoxon test *p* = 0.0003) (Fig. 1).

**Figure 2:**
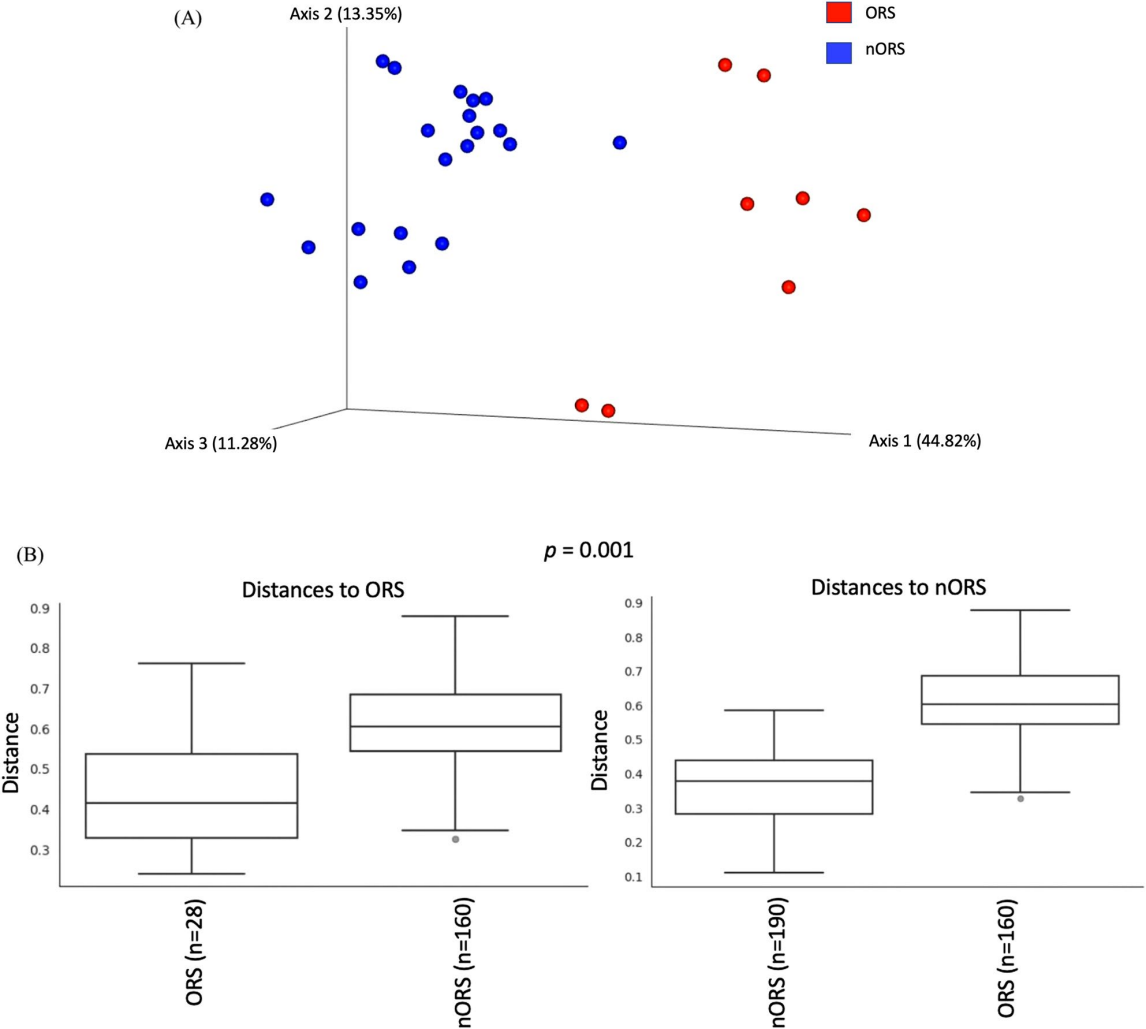
(**A**) Principal coordinate analysis (PCoA) plot with weighted UniFrac distance. Blue and red dots indicate samples from odontogenic rhinosinusitis and nonodontogenic rhinosinusitis, respectively. (**B**) Boxplot of weighted UniFrac distance within each sample and between odontogenic and nonodontogenic rhinosinusitis samples*.*

The visualization of PCoA of samples using QIIME2 based on the weighted UniFrac distance were revealed in Fig. 2A. Each dot represents an individual bacterial community—incorporating both presence/absence and relative abundance of bacterial community members—compared with all other individuals (closer = more similar; farther apart = more dissimilar)^19^. Red and blue dots indicate the odontogenic and nonodontogenic rhinosinusitis samples, respectively. Compared with the dispersed nonodontogenic rhinosinusitis samples, the odontogenic rhinosinusitis samples were more closely clustered on the PCoA plot.

The weighted UniFrac distance within each sample group and between the odontogenic and nonodontogenic groups were analyzed and illustrated. Permutational multivariate analysis of variance (PERMANOVA) revealed a significant difference between both groups (*p* = 0.001) (Fig. 2B).

**Figure 3:**
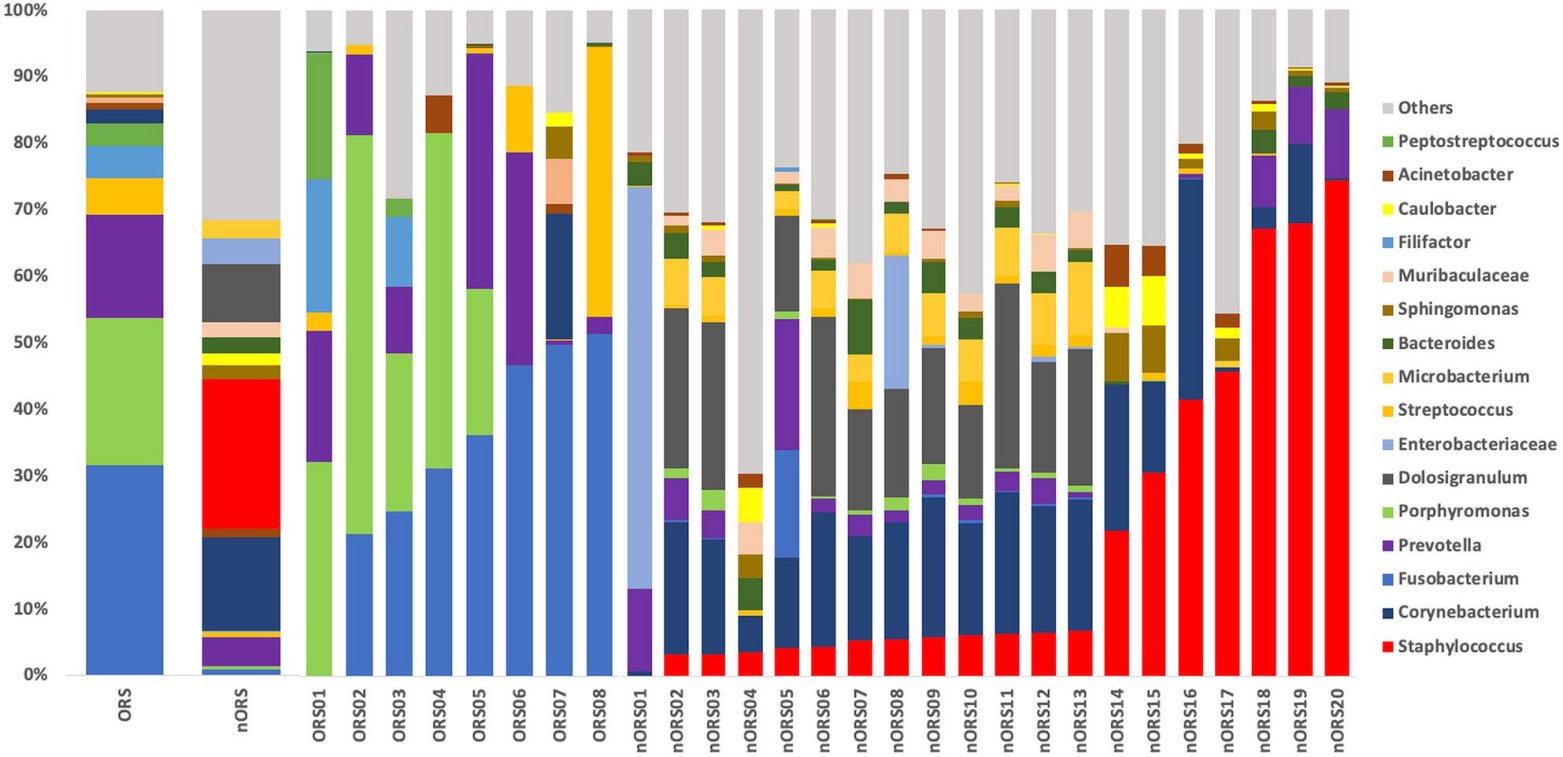
16S rRNA gene-based bacterial community composition and abundance data for middle meatus samples collected from patients with odontogenic rhinosinusitis (ORS) and nonodontogenic rhinosinusitis (nORS). Taxa having less than 1% abundance were annotated as others.

The microbiota distribution (relative operational taxonomic unit composition) at the genus level in both odontogenic and nonodontogenic rhinosinusitis patients were illustrated. We mapped from the 16S rRNA sequencing data to taxonomy using the feature-classifier module of QIIME2, with the SILVA database as reference. Read counts of each feature at the genus level were calculated, and features with < 50 reads were filtered out. After filtering, we calculated relative abundance as a ratio of read counts in each feature to total reads in each sample. The results revealed that the microbiota distribution was different between odontogenic and nonodontogenic rhinosinusitis patients (Fig. 3). *Fusobacterium*, *Porphyromonas*, and *Prevotella* were dominant in the samples from the odontogenic rhinosinusitis group; by contrast, *Staphylococcus* and *Corynebacterium* were dominant in the samples from the nonodontogenic rhinosinusitis group (Fig. 3).

To discover DAMs between the samples, we filtered genera with a log2-fold change of ≥ 2 or ≤ − 2, *p* value of < 0.05, and base mean read count of > 100 using DEseq2. The genera with top differential abundance at the genus level were summarized (supplemental Table 1). A positive log2-fold change indicated that the proportion of a specific bacterium from the odontogenic rhinosinusitis samples was more than that from the nonodontogenic samples, and a negative log2-fold change indicated that the proportion of a specific bacterium from the nonodontogenic rhinosinusitis samples was more than that from the odontogenic samples. *Porphyromonas*, *Fusobacterium*, *Campylobacter*, *Prevotella,* and *Streptococcus* were significantly dominant in the odontogenic rhinosinusitis samples, whereas, *Staphylococcus, Corynebacterium, Bacteroides, Microbacterium*, and *Dolosigranulum* were dominant in the nonodontogenic rhinosinusitis samples.

**Figure 4:**
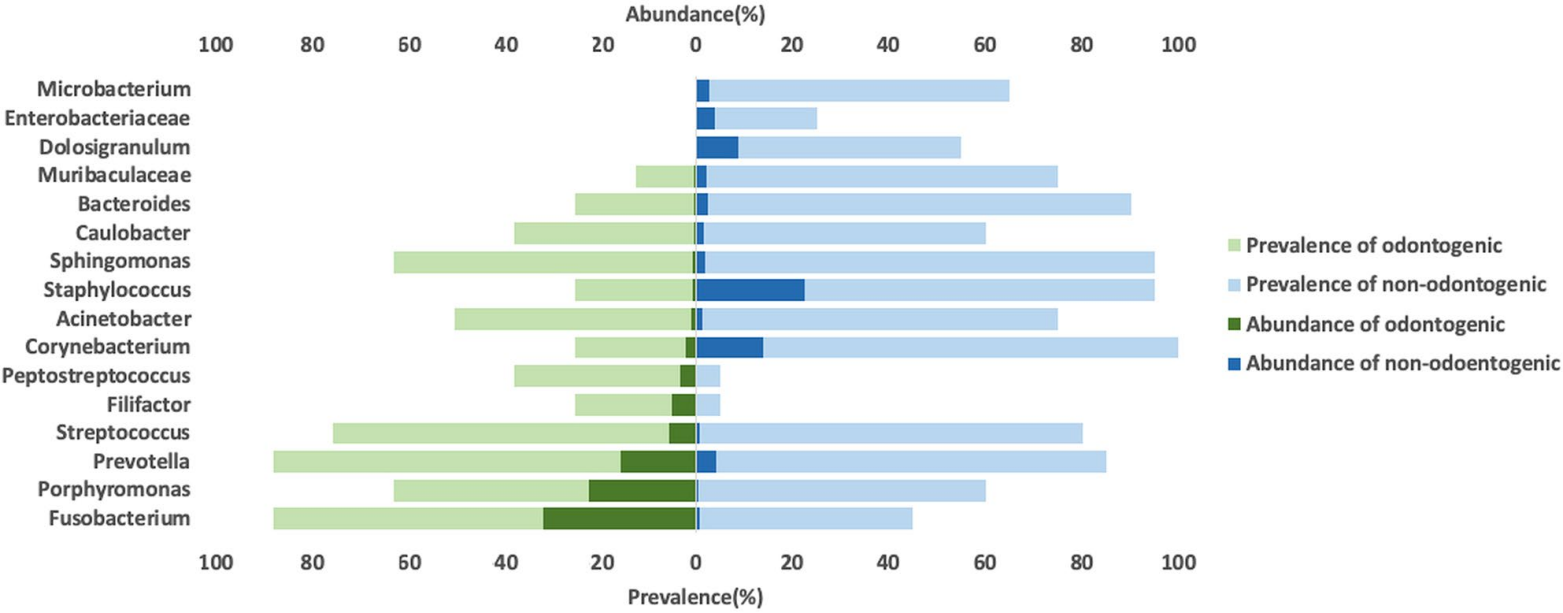
Relative abundance and prevalence based on 16S sequencing data in odontogenic and nonodontogenic rhinosinusitis samples. Abundance was calculated as the ratio of the number of read counts in each feature to total reads in each sample. Prevalence was calculated as the ratio of the number of samples that had a specific microbiome to the number of total samples in different groups. Only taxa with an average proportion of more than 1% in samples were represented in this figure. Green represents odontogenic rhinosinusitis, and blue represents nonodontogenic rhinosinusitis.

The genera with ≥ 1% abundance in either group were collected to assess the most prevalent and relatively abundant microbiomes (Fig. 4). *Fusobacterium* and *Staphylococcus* had the highest abundance in the odontogenic group and nonodontogenic rhinosinusitis group, respectively. Furthermore, *Fusobacterium* and *Corynebacterium* had the highest prevalence in the odontogenic group and nonodontogenic rhinosinusitis group, respectively. In addition, *Microbacterium, Enterobacteriaceae,* and *Dolosigranulum* were rare, occurring only in the nonodontogenic group; *Peptostreptococcus* and *Filifacter* occurred only in the odontogenic group.

**Figure 5:**
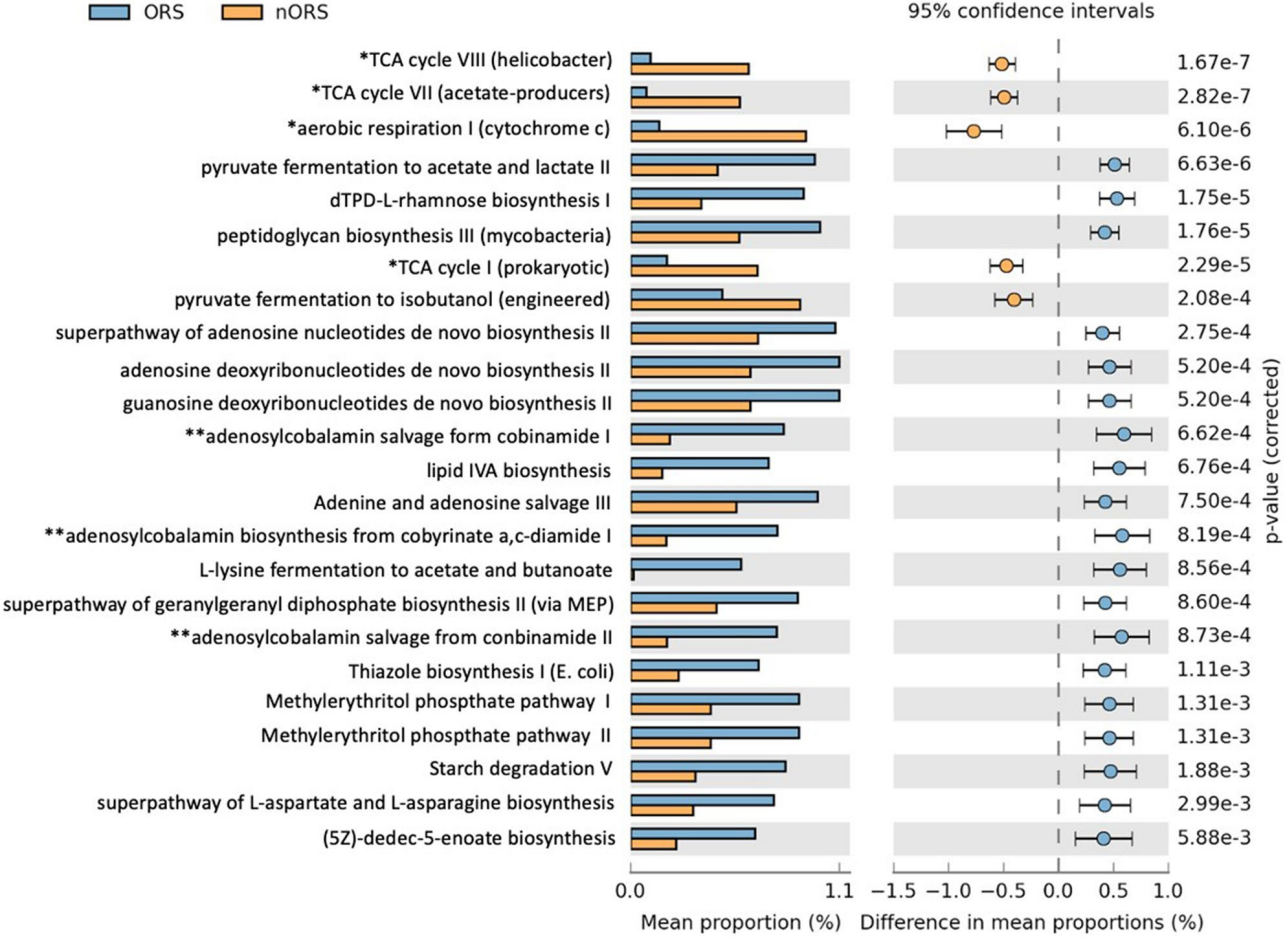
Functional analysis of the microbiota in ORS and nORS samples. *TCA related pathway and aerobic respiration pathway are dominant in nORS group. **Adenosylcobalamin biosynthesis related pathways are dominant in ORS group.

The functional analysis of microbiome between ORS and nORS samples were performed by PICRUSt2. As shown in Fig. 5, results demonstrated that the abundances of several functional categories showing significant difference between two groups at a false discovery rate of 5%. Several aerobic-related categories were notably listed. We observed a decrease in the abundance of microbes involved in TCA cycle and aerobic respiration, and increase in adenosylcobalamin biosynthesis related pathways (adenosylcobalamin biosynthesis from cobyrinate a,c-diamide I, adenosylcobalamin salvage from cobinamide I, andadenosylcobalamin salvage from cobanamide II) in ORS group as compared to nORS group.

**Figure 6:**
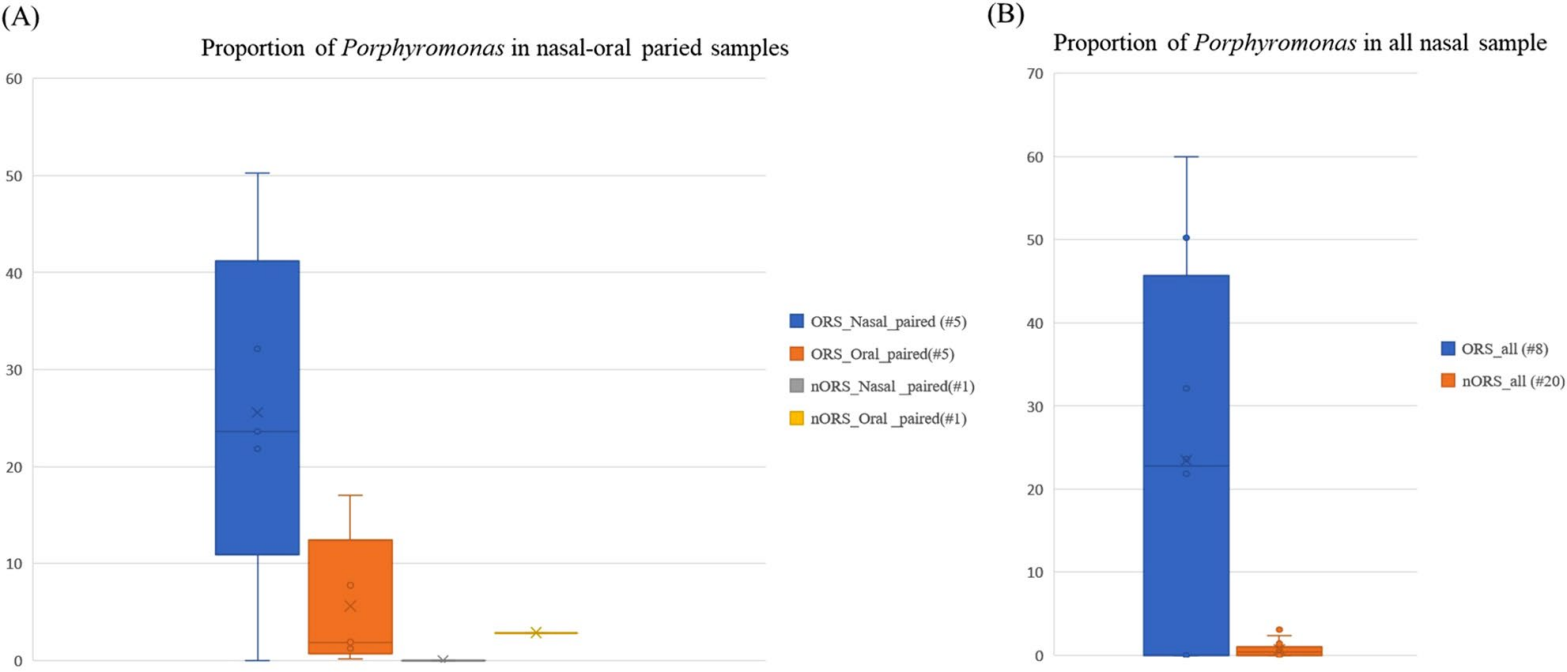
Boxplot of *Porphyromonas* proportion in (**A**) nasal-oral paired samples (**B**) all nasal samples in ORS group and nORS group.

For observing the microbiome association between nasal and oral, 5 ORS and 1 nORS nasal-oral paired samples were collected for further analysis. Three red-complex bacteria were mainly focused, *Bacteroides forsythus*, *Porphyromonas gingivalis*, and *Treponema denticola*, which are implicated in severe forms of periodontal diseases^20^. Due to the taxonomic resolution of targeting of 16S variable regions with short-read sequencing, here we focused on genus level. Analysis results show that *Bacteroides* and *Treponema* were very low abundance among these paired samples (< 0.1%). However, *Porphyromonas* were high abundance in both ORS nasal and oral samples, and low abundance in nORS samples (Fig. 6A). Interestingly, in all of our nasal samples, *Porphyromonas* also shows high abundance in ORS samples and low abundance in nORS sample (Fig. 6B), which may imply potential associations between nasal and oral in odontogenic rhinosinusitis patients.

### Correlations between relative abundance of bacteria

**Figure 7:**
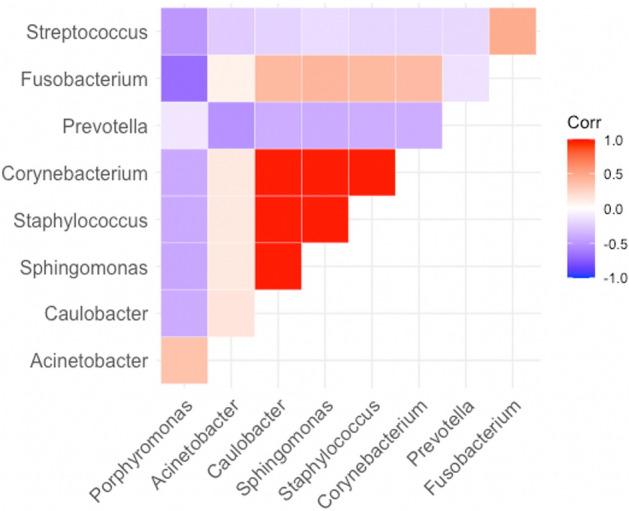
Correlation between different bacteria genera from odontogenic rhinosinusitis samples. Red and blue indicate positive and negative correlations from 16S data, respectively.

The Pearson’s correlation of 16S sequencing data was evaluated, with filtering at a coefficient *p* value of 0.05 (Fig. 7). Significant positive and negative correlations were observed among different bacteria from the odontogenic rhinosinusitis samples. *Staphylococcus* was highly associated and positively correlated with *Corynebacterium*, *Sphinogomonas,* and *Caulobacter*; similarly, *Streptococcus* was highly associated and positively correlated with *Fusobacterium*. By contrast, *Prophyromonas* exhibited a highly negative correlation with *Staphylococcus, Streptococcus*, and *Fusobacterium*.

## Discussion

This is the first study to analyze microbiome diversity between patients with odontogenic and nonodontogenic rhinosinusitis as well as the correlation between the involved bacteria. Regarding alpha diversity, the Shannon diversity index revealed a significant microbiome difference between the two groups (Wilcoxon test *p* = 0.0003). PCoA of beta diversity, derived by calculating the Bray–Curtis distance of each sample, also revealed a significant difference between both groups (Wilcoxon test *p* = 0.001). The odontogenic rhinosinusitis group had significantly high abundance and prevalence of anaerobic bacteria such as *Fusobacterium, Prevotella,* and *Porphyromonas*. Notably, Pearson’s correlation analysis revealed significantly positive and negative correlations among different bacteria from the odontogenic rhinosinusitis samples. *Staphylococcus* was highly associated and positively correlated with *Corynebacterium* but negatively correlated with *Prophyromonas*.

Odontogenic rhinosinusitis, originating from odontogenic infection or iatrogenic injury from dental or oral procedures, is distinct from other types of rhinosinusitis^13^. Most odontogenic rhinosinusitis cases are unilateral and localized. Radiographic features of odontogenic rhinosinusitis most commonly reveal unilateral maxillary sinusitis^4^. Saibene et al. reported that up to 80% of odontogenic rhinosinusitis cases were unilateral^1^. In our study, all odontogenic rhinosinusitis cases were unilateral (8 of 8), and this frequency was significantly higher than that in the nonodontogenic rhinosinusitis group (9/20); this rate is similar to that of a previous study^1,2,4,5^. Although two-dimensional periapical and panoramic radiographs are helpful in the diagnosis of periapical lesions, they are not sufficient to clearly evaluate upper maxillary areas compared with 3D techniques such as CT^4,10,21^. Notably, Longhini et al. reported that up to 86% of odontogenic rhinosinusitis cases may be missed when dental radiographs are used for diagnosis^22^. Therefore, CT is considered the gold standard for the diagnosis of odontogenic rhinosinusitis owing to its high resolution and its ability to discern bone and soft tissue inflammation in both the sinus and dental areas. In our study, periapical radiolucency over the ipsilateral maxillary sinusitis side was revealed by CT in every odontogenic rhinosinusitis case; conversely, no periapical lesion was observed in the nonodontogenic rhinosinusitis group.

Several factors have been reported to be associated with CRS, including anatomic abnormalities, immune dysfunction, host genetics, and microbial dysbiosis^15,23^. Odontogenic rhinosinusitis is usually diagnosed when a CT scan indicates a bone defect in the maxillary sinus floor, and odontogenic rhinosinusitis is typically associated with an infection of dental origin^24^. Through metagenomics analysis, Lucas et al. reported that anaerobic microbiota plays a crucial virulence role in CRS; they also revealed that a mucin-degrading anaerobic microbial consortium, composed of *Prevotella*, *Fusobacterium*, and *Streptococcus*, enhances the virulence of CRS–related *S. aureus*^25^. Notably, mucin-degrading anaerobes and *Porphyromonas* have been previously isolated from dental plaque and biofilm^26^. In our study, anaerobic microbiota as *Porphyromonas*, *Prevotella*, *Fusobacterium*, and *Streptococcus* are also significant dominant in odontogenic rhinosinusitis group. Therefore, the mucin-degrading bacteria derived from odontogenic lesions may induce pathogenicity in nasal commensal *S. aureus*, thereby causing mucosal inflammation, possibly followed by immune dysregulation and eventually CRS.

Interestingly, when comparing the functional categories of the bacterial OTUs by using PICRUSt2^17^, we observed a decrease in the abundance of microbes involved in several TCA cycle related metabolic pathways in ORS group (mean proportion difference > 0.5 as compared to nORS group), which is known much important for aerobic growth. An increase of pathways involved in ORS group were found mainly in the vitB12 biosynthesis pathways that was reported significantly decreased in the oral microbiota filled with elevated partial pressures of oxygen during diving^27^.

Herein, significance analysis of functional categories in the OTUs recognized that anaerobic bacteria play a critical role in odontogenic rhinosinusitis. Saibene et al. found anaerobes in 14% of patients with odontogenic sinusitis as well as sinonasal complications of dental disease or treatment (SCDDT) compared with the rate of 7% in patients with CRS with nasal polyp (CRSwNP)^1^. In addition, according to culture results, dominant anaerobes isolated from SCDDT were *Dialister pneumosintes*, *Peptostreptococcus* spp*.*, *Prevotella dentalis*, *Fusobacterium nucleatum*, *Propionibacterium acnes, Peptostreptococcus* spp., and *Prevotella oralis*^1^. Otherwise, in the CRSwNP group, *Staphylococcus* was the dominant aerobe^1^. In our study, anaerobic bacteria including *Porphyromonas, Fusobacterium, Campylobacter,* and *Prevotella* were significantly dominant in the odontogenic rhinosinusitis samples, whereas *Staphylococcus* had the highest prevalence and abundance in the nonodontogenic rhinosinusitis samples. Our results are consistent with those of previous studies. However, Saibene et al. reported that 60% of control samples failed to yield any bacterial growth^1^. Culture-dependent methods cannot distinguish subtle differences between two microbiota or even reveal bacterial interactions. We therefore applied 16SrRNA sequencing, a culture-independent method, to address these two limitations. In our study, significant microbiota diversity was observed between the odontogenic and nonodontogenic rhinosinusitis groups. Regarding alpha diversity, the Shannon diversity index indicated a significant difference in the microbiome (Wilcoxon test *p* = 0.0003). PCoA of beta diversity, derived by calculating the Bray–Curtis distance of each sample, also revealed a significant difference (Wilcoxon test *p* = 0.001).

In the odontogenic rhinosinusitis group, *S. aureus* was positively correlated with *Corynebacterium;* similarly, *Streptococcus* was highly associated and positively correlated with *Fusobacterium.* Several studies have reported that *Corynebacterium* often co-occurs with *S. aureus*^28,29^. Coincubation of *S. aureus* with nasal isolated *Corynebacterium striatum* strains affected the Agar system and induced the expression of adhesins, with roles in nasal colonization^30^. Furthermore, Guo et al. observed adhesin-mediated bacterial interspecies interactions between *Streptococcus mutants* and *F. nucleatum* ssp. *polymorphum*^31^. By contrast, in odontogenic rhinosinusitis group, *Prophyromonas* exhibited a highly negative correlation with *Staphylococcus, Streptococcus,* and *Fusobacterium.* Wang et al. demonstrated that *Streptococcus cristatus* inhibits the expression of adhesins in *Prophyromonas gingivitis*, the major causative pathogen of adult periodontitis, and interrupts biofilm formation in *P. gingivitis*^32^. A negative correlation between the distribution of *P. gingivitis* and *S. cristatus* has also been reported^32^. The presence of *Corynebacterium/Fusobacterium/Prophyromonas* may, therefore, be a useful predictor for the tendency to carry specific pathogens.

Periodontitis is an inflammatory disease of the gingiva, accompanied by supportive connective tissues loss, such as the alveolar bone and periodontal ligament^33^. According to a longitudinal follow-up study using national health-Screening cohort by Byun et al., CRS patients were more likely to receive the diagnosis of periodontitis^34^. Except the close relationship between CRS and periodontitis in big data research results, in pathogenesis mechanism, periodontitis and CRS were both chronic inflammation disease. The main possible pathogenesis of periodontitis were interactions between different bacteria, and environmental factors which is similar to CRS^34,35^. In patients with periodontitis, interactions between pathogen-associated molecular patterns and pattern recognition receptors by bacteria can initiate inflammatory reaction^34,36^. *Actinobacillus actinomycetemcomitans, Porphyromonas gingivalis, Prevotella intermedia*,and *Tannerella forsythensis* were discovered as the the most dominant anaerobic bacteria involved in periodontitis^37^. Furthermore, *Bacteroides forsythus*, *Porphyromonas gingivalis*, and *Treponema denticola* were mentioned as the” red-complex” bacteria which implicated in severe forms of periodontal diseases^20^. These bacteria play an important role to onset the periodontitis and then induce immunologic and pathogenic reactions by producing various cytokine and biological elements^34^. Furthermore, on dental surface, these bacteria proliferate and form the plaque which is an agglomeration of biofilm. Similarly, bacterial biofilm also plays important role in the formation of CRS^38^. In 2004, Palmer et al. first reported the existence of biofilms on the sinus mucosa of patients with recalcitrant CRS^39^ and numerous studies have subsequently supported this theory^39,40^. In our study, *Porphyromonas* and *Prevotella* are significant dominant bacteria genus in odontogenic rhinosinusitis groups which is compatible with above previous study. Furthermore, in nasal-oral pairing samples, *Porphyromonas* is also dominant in ORS group comparing with nORS group (Fig. 6A).

This study has several limitations. First, odontogenic rhinosinusitis is rare compared with other rhinosinusitis subtypes; therefore, we were able to include only eight patients with odontogenic rhinosinusitis. Second, fungal microbiota was not included in this study. The evaluation of both fungal microbiota and oral microbiome can enable us to understand the microenvironment of odontogenic rhinosinusitis. Accordingly, future research should include more patients and evaluate the internal transcribed spacers of fungal microbiota as well as oral microbiomes to comprehensively elucidate odontogenic rhinosinusitis.

## Conclusion

To the best of our knowledge, this is the first study to use next generation sequencing methods (NGS) to investigate the difference in bacterial microbiota between patients with odontogenic rhinosinusitis and those with nonodontogenic rhinosinusitis. Microbiota dysbiosis and predominance of anaerobic bacteria were significant in patients with odontogenic rhinosinusitis compared with those with nonodontogenic rhinosinusitis. Furthermore, the correlation between different bacteria in odontogenic rhinosinusitis may be associated with dental biofilms and mucin degradation. Therefore, odontogenic rhinosinusitis should be considered distinct from other nonodontogenic rhinosinusitis.

## Supplementary Information

Supplementary Table 1.

## Data Availability

The datasets generated and analyzed during the current study are available in the NCBI Sequence Read Archive repository with bioproject accession number PRJNA847388, https://dataview.ncbi.nlm.nih.gov/object/PRJNA847388.
