# Supplementary material for: Microbiota dysbiosis in odontogenic rhinosinusitis and its association with anaerobic bacteria
Source: Sci Rep. 2022 Dec 5;12:21023. doi: 10.1038/s41598-022-24921-z (PMC9722704; doi:10.1038/s41598-022-24921-z)
Supplement: Supplementary file 1 — Supplementary Table 1. [file 41598_2022_24921_MOESM1_ESM.docx]

**Supplement Table 1**. The top differentially abundant genus between odontogenic and non-odontogenic rhinosinusitis samples.

| **Taxonomy** | **BaseMean read counts** | **log2FoldChange** (ORS / nORS) | ***p* value** |
| --- | --- | --- | --- |
| *Eubacterium_brachy* | 117.19232 | 8.413478 | 3.12E-19 |
| *Parvimonas* | 197.70475 | 6.394789 | 1.57E-10 |
| *Porphyromonas* | 3615.13017 | 5.767386 | 4.03E-07 |
| *Fusobacterium* | 5142.53741 | 5.427656 | 1.94E-05 |
| *Streptococcus* | 1149.08830 | 2.907013 | 0.00382799 |
| *Campylobacter* | 235.31235 | 3.117782 | 0.00437489 |
| *Prevotella* | 3797.90419 | 2.169548 | 0.02740006 |
| *Dolosigranulum* | 2718.79230 | -11.477814 | 2.78E-19 |
| *Microbacterium* | 881.63798 | -9.852788 | 1.21E-17 |
| *Muribaculaceae* | 686.19463 | -8.483010 | 1.71E-16 |
| *Escherichia.Shigella* | 385.90392 | -8.065615 | 2.58E-16 |
| *Clostridia* | 265.30084 | -7.211313 | 2.75E-15 |
| *Lachnospiraceae* | 249.26390 | -6.441192 | 2.83E-14 |
| *Lachnospiraceae_NK4A136* | 118.80986 | -6.957537 | 8.23E-14 |
| *Lactobacillus* | 266.98430 | -6.079383 | 1.33E-10 |
| *Rhodococcus* | 139.34122 | -7.187936 | 1.72E-10 |
| *Faecalibacterium* | 241.49809 | -5.052518 | 1.01E-07 |
| *Enterobacteriaceae* | 149.08393 | -7.285491 | 1.31E-07 |
| *Cutibacterium* | 292.89860 | -4.712488 | 1.89E-07 |
| *Bacteroides* | 810.45507 | -4.694151 | 4.30E-07 |
| *Paracoccus* | 108.26300 | -4.951670 | 4.78E-07 |
| *Peptoniphilus* | 276.81186 | -5.119637 | 8.09E-07 |
| *Bradyrhizobium* | 219.80371 | -4.823791 | 1.12E-05 |
| *Staphylococcus* | 7192.76546 | -4.736152 | 1.30E-05 |
| *Sphingomonas* | 667.20597 | -3.869440 | 1.71E-05 |
| *Chloroplast* | 141.96586 | -4.952741 | 2.01E-05 |
| *Caulobacter* | 542.00697 | -4.981967 | 2.59E-05 |
| *Corynebacterium* | 4533.49634 | -3.587423 | 0.00031266 |
| *Chitinophagaceae* | 229.53386 | -4.286923 | 0.00059806 |
| *Phascolarctobacterium* | 106.02765 | -2.419997 | 0.02896464 |

ORS: odontogenic rhinosinusitis; nORS: non-odontogenic rhinosinusitis
